# An Infant Formula with Partially Hydrolyzed Whey Protein Supports Adequate Growth and Is Safe and Well-Tolerated in Healthy, Term Infants: A Randomized, Double-Blind, Equivalence Trial

**DOI:** 10.3390/nu12072072

**Published:** 2020-07-13

**Authors:** Jean-Charles Picaud, Barbara Pajek, Malgorzata Arciszewska, Izabela Tarczón, Joaquin Escribano, Rocio Porcel, Thomas Adelt, Elly Hassink, Anneke Rijnierse, Marieke Abrahamse-Berkeveld, Bartosz Korczowski

**Affiliations:** 1 Hospices Civils de Lyon; Neonatal Intensive Care Unit, Hôpital de la Croix Rousse, 69004 Lyon, France; 2 Univ Lyon, CarMen laboratory, INSERM, INRA, Université Claude Bernard Lyon 1, 69310 Pierre Bénite, France; 3 NZLA Michalkowice Jarosz i Partnerzy Spolka Lekarska, 41-103 Siemianowice-Slaskie, Poland; barbarapajek@interia.pl; 4 Poliklinika Ginekologiczno-Poloznicza Sp. z o.o. Sp.k, 15-435 Bialystok, Poland; malgorzata@arciszewska.eu; 5 Specjalistyczna Poradnia Medyczna Przylądek Zdrowia, 31-589 Kraków, Poland; lekarz@przyladekzdrowia.pl; 6 Hospital Universitari Sant Joan de Reus, 43204 Reus, Spain; jescribano@grupsagessa.com; 7 Hospital Quirónsalud Barcelona, 08023 Barcelona, Spain; rocio.porcel@quironsalud.es; 8 Paediatric Practice, 49565 Bramsche, Germany; thomasadelt@yahoo.de; 9 Danone Nutricia Research, 3584 CT Utrecht, The Netherlands; elly.hassink@danone.com (E.H.); anneke.rijnierse@danone.com (A.R.); marieke.abrahamse@danone.com (M.A.-B.); 10 Department of Pediatrics and Pediatric Gastroenterology, College of Medical Sciences, University of Rzeszów, 35-302 Rzeszów, Poland; korczowski@op.pl

**Keywords:** safety, partially hydrolyzed formula, tolerance, infant growth, prebiotic

## Abstract

The current study evaluates the safety and tolerance of a partially hydrolyzed whey protein-based infant formula (PHF) versus an in intact cow’s milk protein formula (IPF). Breastfed infants were included as a reference group. In a multi-country, multicenter, randomized, double-blinded, controlled clinical trial, infants whose mothers intended to fully formula feed were randomized to PHF (*n* = 134) or IPF (*n* = 134) from ≤14 days to 17 weeks of age. The equivalence analysis of weight gain per day within margins of +/−3 g/d (primary outcome), the recorded adverse events, growth and gastro-intestinal tolerance parameters were considered for the safety evaluation. Equivalence of weight gain per day from enrolment until 17 weeks of age was demonstrated in the PHF group compared to the IPF group (difference in means −1.2 g/d; 90% CI (−2.42; 0.02)), with estimated means (SE) of 30.2 (0.5) g/d and 31.4 (0.5) g/d, respectively. No significant differences in growth outcomes, the number, severity or type of (serious) adverse events and tolerance outcomes, were observed between the two formula groups. A partially hydrolyzed whey protein-based infant formula supports adequate infant growth, with a daily weight gain equivalent to a standard intact protein-based formula; it is also safe for use and well-tolerated in healthy term infants.

## 1. Introduction

Exclusive human milk feeding is uniquely suited to, and is the preferred mode of feeding for, term infants. In cases where (exclusive) breastfeeding is not possible, infants should receive specialized infant milk formulas aimed at providing their nutritional requirements, with functional properties close to those of human milk and compliant with stringent regulations [1].

Human milk contains bioactive and immunological components and has been shown to protect against infections and allergic disease in infancy [2,3]. Evidence-based guidelines recommend that a (partially) hydrolyzed formula (PHF), in place of a standard cow’s milk protein-based formula, is provided to infants with an increased risk for allergies who are not (exclusively) breastfed in order to lower their risk of allergic sensitization [4,5,6]. Previously, the impact of a specific PHF supplemented with oligosaccharides versus intact protein formula (IPF) on immune outcomes in infants at high risk of allergic disease was evaluated [7]. Although the cumulative incidence of eczema in the first year of life was not reduced (primary outcome), positive immune effects (i.e., a reduction in cow’s milk-specific IgG1 levels) were observed, in line with previous observations that PHF and oligosaccharides influence regulatory T cells [8,9,10]. However, as concluded in recent systematic reviews and meta-analyses [11,12], the concept that such dietary exposures may influence infants’ immune development needs confirmation in separate, well-designed studies, preferably with longer-term follow-ups in order to assess the relevance to health outcomes in later life.

The hydrolysis of protein sources aims to reduce their allergenicity in order to prevent allergic sensitization. However, the bioavailability of nitrogen and other nutritional components of infant milk formula might be affected by the hydrolysis of protein sources [13,14], i.e., leading to imbalances in plasma amino acid concentrations [15]. Moreover, compared to standard intact cow’s milk formula, (extensively) hydrolyzed formulas have been associated with differences in infant growth patterns [16,17]. Hence, as a first step, the nutritional adequacy and safety of (partially) hydrolyzed infant formulas need to be confirmed [1,18]. The aforementioned PHF, supplemented with specific prebiotic mixtures, was shown to support adequate growth according to the WHO growth standards, and it was found to be safe and well-tolerated in infants [7,19]. However, a more stringent, specifically designed clinical evaluation is required in order to confirm its safety and suitability, including a head-to-head comparison to a standard intact cow’s milk protein formula [20,21].

In the current study, the primary objective was to evaluate the growth, defined as daily weight gain, of infants who were fed a partially hydrolyzed whey protein-based infant formula (PHF), compared with those fed a standard infant formula containing intact protein (IPF). Several secondary objectives, related to other anthropometric measures, tolerance, stool characteristics and adverse events, were evaluated. As a reference, a group of breastfed infants was included.

## 2. Materials and Methods

### 2.1. Participating Centers

A total of 15 study centers in six countries participated in the study, including Poland (5 centers: Specialistyczna Poradnia Medyczna Przyladek Zdrowia, Poliklinika Ginekologiczno-Poloznicza, Korczowski Bartosz Gabinet Lekarski, Polmed Jan Mazela Instytut Microekologii, NZLA Michalkowice Jarosz i Partnerzy Spolka Lekarska), Germany (2 centers: Pediatric Practice of Dr. Thomas Adelt, Vivantes Clinic of Obstetric Medicine, Berlin-Neukoelnn), the Netherlands (1 center; Gelre Ziekenhuizen locatie Apeldoorn), France (2 centers; Hospices Civils de Lyon, Hopital de la Croix Rousse and Groupement des Hopitaux de l’Institut Catholique de Lille, Hopital Saint Vincent de Paul), Spain (4 centers: Hospital Quironsalud Barcelona, Hospital General Universitari D’Elx, Hospital Universitari de Tarragona Joan XXII, Hospital Universitari Sant Joan de Reus) and Finland (1 center: Turku University Hospital, Department of Paediatrics). The approval of the independent local Ethical Review Boards was obtained by all participating centers. The study was conducted in compliance with the local laws and regulations of the countries in which the study was performed, according to the principles of the Declaration of Helsinki and the ICH-GCP principles. This trial was registered at www.clinicaltrials.gov with identifier NCT03062761. Details of the study protocol are provided as Supplementary Information.

### 2.2. Subjects and Study Design

Healthy term infants, with a gestational age between 37 weeks and 42 weeks, postnatal age ≤14 days, a birth weight between the 10th and 90th percentile according to the Intergrowth Standards [22] or available local growth chart, a head circumference within normal range for age and sex (within 2 SD according to local or WHO Growth Standards [23]) and whose mothers intended to fully formula-feed or fully breastfeed, were eligible for participation. Exclusion criteria were defined as the following: special dietary needs other than standard infant formula, infant illnesses that could interfere with the study, maternal medical conditions (including during pregnancy) that could interfere with the study (e.g., eclampsia or gestational diabetes), maternal or infant participation in any other study involving products or potential inability to provide protocol compliance. Before enrolment, written informed consent from all parents/guardians was obtained. The study was designed as a randomized, controlled, double-blind, parallel group, multi-country, (growth) equivalence trial. A dedicated clinical studies supplies manager at the sponsor coded the formulas; investigators and parents were blinded to the coding of the formulas. The randomization sequence was generated using sex (male/female) and country as strata (PLAN procedure in SAS statistical software; Enterprise Guide version 4.3) by a statistician from Danone Nutricia Research who had no other involvement in the study conduct. The generated randomization sequence was uploaded to a central interactive web-response system (IWRS). After enrolment and input of relevant subject data by the investigator, formula-fed infants were randomly assigned a unique code referring to a product tin on-site by the IWRS, containing 1 of 2 study formulas. The investigator obtained the product code and provided the applicable tins to the subject. In the case of twins, one of the twin infants was randomized and the other was assigned to the same product. As a reference, a group of breastfed infants was included. Breastfed infants meeting all inclusion criteria and whose mother had the intention to breastfeed exclusively until 17 weeks of age were eligible for participation. During the study, infants were fully formula-fed or fully, but not exclusively [24], breast-fed, since it was allowed to provide water, tea or rehydration solutions, drops or syrups (vitamins, minerals, medicines) to the infants.

### 2.3. Study Products

The intervention formulas were standard, commercially available infant formulas, provided in 400 g tins, compliant with Directive 2006/141/EC and manufactured according to the standards (ISO 22000) and (Table 1). Given the potential impact on bioavailability [13,14], the partially hydrolyzed whey protein-based infant formula (PHF) had a higher protein level versus the standard intact protein infant formula (PHF; 2.3 g/100 kcal vs. IPF; 2.0 g/100 kcal), which was mainly compensated for by a lower carbohydrate level (10.9 g/100 kcal vs. 11.3 g/100 kcal) to maintain an isocaloric content of 66 kcal/100 mL. A specific prebiotic mixture of short-chain galacto-oligosaccharides (90%) and long-chain fructo-oligosaccharides (10%) was present in both formulas (scGOS/lcFOS, 0.8 g/100 mL).

### 2.4. Measurements

Daily weight gain (g/d) from enrolment until 17 weeks of age was the primary outcome measure of the study. Secondary outcomes comprised measurements of length, head circumference, mid-upper arm circumference, tolerance parameters, formula intake, plasma parameters and adverse events.

Infants had an enrolment (baseline) visit ≤14 days of age (V 1), followed by visits at 4 (V2), 8 (V3), 13 (V4) and 17 (V5) weeks of age. At 19 weeks of age, parents were contacted by phone to follow up on any (serious) adverse events, including (changes in) use of medication or nutritional supplements which started before the last clinic visit.

At the enrolment visit, infant characteristics and demographic information were collected. Anthropometric parameters were measured at enrolment (baseline) and at each visit thereafter. Infants were weighed twice, naked, on the same type of calibrated electronic scales (Kern MBC 20K10M, Kern & Sohn GmbH, Balingen, Germany). A standard measuring board was used to measure the supine length of infants twice. Head circumference and mid-upper arm circumference was measured in duplicate with a non-stretchable measuring tape (recording whether this was done for the left or right arm) in duplicate. A third measurement was obtained in case the anthropometric measures deviated substantially (>20 g for weight and >5 mm for length, head and arm circumference), averaging the measurements which were closest together as the final outcome measurement.

During the 7-day period preceding each visit, gastrointestinal symptoms, daily study product intake and stool characteristics were recorded by the parents in electronic diaries. At each visit, the investigator discussed the entries in the diaries to ensure the information was complete and verified for plausibility. Severity of regurgitation and vomiting, return of milk into the mouth with or without force, respectively, was indicated by a combination of daily frequency and number of days on which it was recorded. Regurgitation severity categories were defined as the following: one day with regurgitation (occasionally), one day with 3 or more regurgitations (commonly), at least 2 or 3 days with 3 or more regurgitations (frequently). Severity of vomiting was defined as follows: one day with vomiting or more than one day of vomiting. A 4-point scale using pictures (watery, soft, formed, hard) according to the “Amsterdam” stool form scale was used to score stool consistency for each stool passed [25]. During the study, investigators documented (serious) adverse events (AEs) at each visit. Their onset, duration, relationship with the study product, severity and seriousness and any actions that were taken with their outcomes were recorded. (S)AEs were followed up on by the investigator until a stable situation was reached or until they abated.

At 17 weeks of age, after obtaining additional consent, a venous serum sample was collected. The serum was stored at −20 °C or −80 °C until shipment to Danone Nutricia Research every three months or at the end of the study. The levels of albumin, minerals (calcium, phosphorus, ferritin and magnesium) and blood urea nitrogen were determined as markers of nutritional status in a central laboratory (Reinier Haga Medisch Diagnostische Centrum BV, Delft, The Netherlands).

### 2.5. Statistics

Daily weight gain (g/d) from enrolment until 17 weeks of age was the primary outcome of the study. The objective was to test the hypothesis of equivalence in daily weight gain of the PHF vs. IPF groups in the per-protocol population. In cases in which the two-sided 90% confidence interval (CI) of the difference in means of daily weight gain was contained within the pre-defined equivalence margins of ±3 g/d, equivalence was demonstrated [18]. In order to establish equivalence based on the margin of 3.0 g weight gain per day from the baseline until the age 17 weeks, and assuming a difference between formula groups in weight gain of 0.5 g per day, with a significance level (α) of 5% and a power of 80%, 78 evaluable infants per formula group are required (SAS Institute Inc., Cary, NC, USA). This difference in weight gain was a model-based estimate for the PHF versus IPF groups using the pooled historical clinical data of previous intervention studies. Assuming a drop-out rate (including major protocol violators) of 30%, a total of 224 infants (112 per group) were to be enrolled. An interim analysis included a re-estimation of the sample size and was performed and evaluated by a dedicated team of one statistician and two clinical researchers, who were not further involved in the conduct of the study. This team advised us to increase the sample size to a total of 268 infants (134 per arm), which was implemented. In general, if not otherwise mentioned below, two sample *t*-tests (TT) were used for continuous data, and Mann–Whitney U tests (MW) were used in cases of violation of the normality assumption. Categorical response parameters were analyzed by using chi-square tests (C; Fisher’s exact tests in case sparse cells occurred: FE) or the Miettinen and Nurminen approach.

The parametric growth curves mixed model (PGC) was used to perform the equivalence analyses for weight gain, length gain and head circumference gain. This model uses a second order polynomial curve to describe growth parameter development over time, with the stratification factors (country and sex) and birth weight, and the interaction of group and sex with time, and the quadratic effect of time as fixed effects, and each subject’s intercept, slope and quadratic term as random effects. An additional sensitivity analysis was performed by including gestational age as a covariate. The robustness of the results was evaluated in sensitivity analyses using the arbitrary means model (AMM) at 17 weeks of age, with the same structure of fixed and random effects as in the PGC while using age as a categorical variable (scheduled visit). A model-based comparison between formula and breastfed infants was explored, for growth outcomes only, and the additional covariates of maternal BMI, maternal education and maternal smoking were included. The AMM approach was also used for analysis of the WHO growth standard z-scores [23]. Tolerance outcome evaluations included diary data of subjects per visit period only when at least 3 informative days in that period were available for all subjects treated (AST). Statistical comparisons were performed for PHF versus IPF groups. Stool consistency was evaluated for each subject by calculating the percentage of stools for each consistency score per visit (i.e., number of stools with certain consistency divided by total number of recorded stools). Subsequently, data were summarized per outcome, per group and per visit, resulting in descriptions of %watery, %soft, %formed and %hard stools. A similar approach was taken for stool frequency scores. The stool consistency and frequency scores were analyzed per visit using the Mann–Whitney test. The occurrence of constipation was defined as two or less defecations per week (and if any) associated with hard stools (adapted Rome III criteria). The occurrence of diarrhea was defined as the passage of three or more watery stools per day (adapted from the WHO definition). The occurrence of gastrointestinal (GI) symptoms was analyzed per visit by the Miettinen and Nurminen approach. During the analysis of the serum samples, the ferritin values of subjects with CRP ≥ 10 mg/L were excluded. In the all subjects randomized (ASR) analysis, the data were used for all infants randomized to the study product (or being breastfed). The eligibility of data in the per-protocol (PP) analysis was assessed on visit level. Data were excluded from this analysis when other formulas or solid foods were consumed for >3 days before any visit until 17 weeks of age, respectively. All statistical analyses were performed according to the pre-defined statistical analysis plan constructed before unblinding the dataset, using SAS^®^ (SAS Enterprise Guide 4.3 or higher) for Windows (SAS Institute Inc., Cary, NC, USA).

## 3. Results

### 3.1. Subject Characteristics

**Figure 1:**
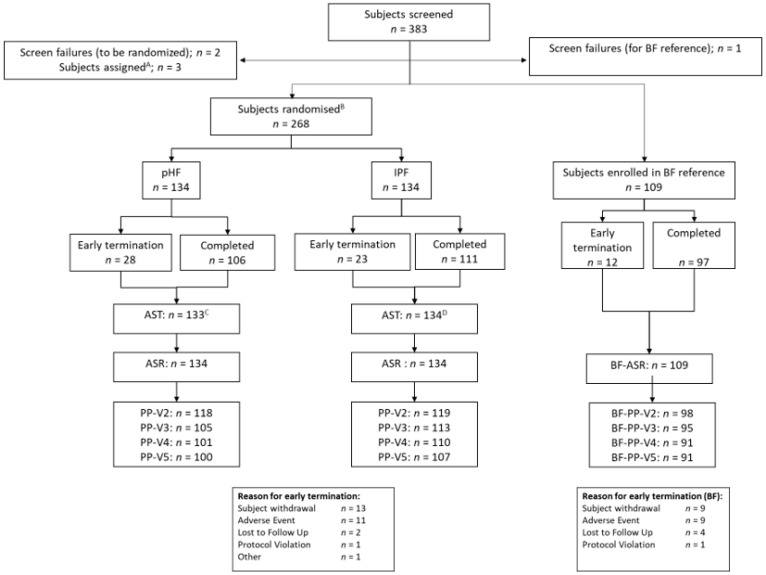
Flow chart of progression of infants during the study. PHF = partially hydrolyzed formula; IPF = intact protein-based formula; BF = breastfed; ASR = all subjects randomized; AST = all subjects treated; PP = per-protocol population assessed per visit (V2 at 4 weeks, V3 at 8 weeks, V4 at 13 weeks and V5 at 17 weeks).^A^ non-randomized part of a twin assigned to the same study product as their sibling (excluded from ASR but included in AST). ^B^ includes three randomized twin subjects; ^C^ *n* = 134 individual subjects minus one subject without study product intake; ^D^ *n* = 134 individual subjects (including three twins, minus three subjects without study product intake).

From April 2017 to June 2018, a total of 383 potential eligible subjects had a screening visit, of which 268 subjects were randomized, 109 infants were part of the breastfed reference group (Figure 1) and three subjects were not eligible.

Four randomized infants were excluded from the AST population since they did not consume the study product. Assigned twin subjects were also part of the AST population. A total of 217 randomized subjects completed the intervention period, resulting in a drop-out rate of 19%; there were 106 completers in the PHF group and 111 completers in the IPF. A total of 97 breastfed infants completed the study up to 17 weeks of age (11% drop-out). The drop-out rate and reasons for early termination were not apparently different between the two formula groups. The predominant reason for early termination in the formula groups was withdrawal from the study or the occurrence of an (serious) adverse event; others were lost-to-follow-up or protocol violation, and one subject in the PHF group was lost for other reasons. No relevant differences were observed in the number or reasons for early termination between formula groups. Demographic data were well balanced and not apparently different between the intervention groups for the PP (Table 2) and ASR populations (data not shown).

### 3.2. Study Product Intake

During the intervention period, the average daily formula intake was similar in both formula groups (Table 3). No apparent differences in volume intake were observed at any timepoint during the study for total daily intake or when daily intake was corrected for body weight.

### 3.3. Growth Outcomes

**Figure 2:**
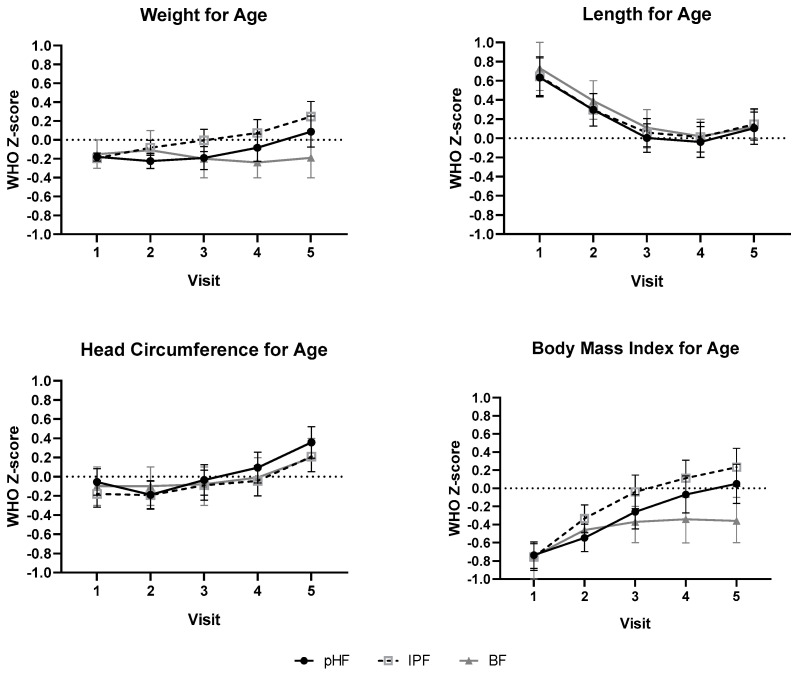
Mean (±95% CI) weight-for-age, length-for-age, head circumference-for-age and BMI-for-age WHO growth standard z-scores per visit for PHF and IPF groups as well as breastfed reference group. V1 before 14 days, V2 at 4 weeks, V3 at 8 weeks, V4 at 13 weeks and V5 at 17 weeks. WHO Z-scores for PHF and IPF are based on arbitrary means model with two groups. Model with (1) fixed effect terms for: group, time (=visit), sex, birth weight and country; (2) interactions for: group by time and sex by time (for a subject) with an unstructured covariance matrix. No statistical differences were observed between the intervention groups. WHO Z-scores for BF reference groups are the non-modeled actual values.

Equivalence of daily weight gain (g/d) from enrolment to 17 weeks of age was demonstrated for the PHF vs. IPF group (mean of 30.2 g/d (95% CI: 29.2, 31.3 g/d) vs. 31.4 g/d (95% CI: 30.4, 32.5 g/d)) in the full PP population, with a difference in estimated means of −1.2 g/d (90% CI (−2.42, 0.02)). The sensitivity analyses of the PP population, including gestational age as an additional covariate, or of the ASR population also demonstrated equivalence (difference in means of −1.2 g/d (90% CI (−2.41, 0.04)) and −1.1 g/d (90% CI (−2.31, 0.10)). Compared to the breastfed reference group (mean of 27.6 g/d (95% CI: 26.4, 28.7 g/d)), formula-fed infants in both intervention groups had a higher mean daily weight gain, resulting in a higher weight at the end of the study period (Table 4). Equivalence in mean difference of daily length and head circumference gain from baseline to 17 weeks of age was demonstrated for both intervention groups in the PP and ASR populations (data not shown). The mean length and head circumference values (Table 4), as well as mean mid-upper arm circumference values (data not shown), were very similar for the formula-fed and breastfed groups of the PP population throughout the intervention period. Compared to the WHO growth standards, the mean weight-for-age and head circumference-for-age z-score values of both intervention groups, as well as the breastfed reference group, lay within the ±0.5SD bandwidth (Figure 2), indicative of adequate infant growth. At the first two visits, the mean length-for-age z-score values were consistently higher for all groups compared to the median of the WHO growth standards and consequently resulted in lower than median BMI z-score values. Over time, both the mean length-for-age and BMI-for-age z-scores in all groups had values close(r) to the median of the WHO growth standard.

### 3.4. Serum Parameters

A voluntary blood sample was obtained at 17 weeks of age from 27 and 38 infants who were randomized to the PHF or IPF groups, respectively, and from 38 breastfed infants as a reference. No statistically significant differences were observed in any of the serum parameters between the intervention groups of the ASR population (Table 5). Most infants had serum values within the adequate ranges (>95% of values), except for the serum calcium level, which was above normal ranges for 22% of formula-fed and 42% of breastfed infants in the ASR population. Compared to the values of breastfed infants as a reference group, higher serum urea levels and lower serum ferritin levels were observed in both intervention groups.

### 3.5. Gastrointestinal Tolerance

Daily stool frequency was not found to be different between randomized groups and remained rather stable during the intervention period, with a median of between 1.2 to 1.9 stools per day (Table 6). In contrast, a substantially higher daily stool frequency was observed in infants in the breastfed reference group, although this decreased over time towards the levels observed in the formula-fed infants. During the intervention period, most infants in the randomized formula groups, as well as breastfed infants, had a stool consistency categorized as “soft stool” (~80%, data not shown). No statistically significant differences in stool consistency were observed between the formula groups, with the exception of a slightly higher percentage of watery stools in the PHF group versus the IPF group at 17 weeks of age (12.7% vs. 8.4%; *p* = 0.022). Interestingly, the breastfed infants had a higher prevalence of watery stools throughout the study period compared to the formula-fed groups (28.8 to 36.9% versus 5.6% to 12.7%, respectively).

During the study period, 2.5% to 7.1% of the randomized infants were reported to have diarrhea (according to the definition of three or more watery stools per day), without significant differences between the formula groups (Table 6). In contrast, the occurrence of diarrhea was substantially higher for breastfed infants based on this definition, declining from 36% of the subjects with diarrhea at 4 weeks of age to 20% at the end of the study period. Constipation, defined as two or less defecations per week, only occurred in one subject from the IPF group at 8 weeks of age.

The percentage of days with regurgitation as well as the severity of regurgitation was not statistically significant different between infants of the PHF and IPF groups throughout the study. Regurgitation was considerably more prevalent across all regurgitation severity categories for the breastfed reference group versus the two formula groups (Table 6). No differences in the occurrence or severity of vomiting was observed for infants in the PHF versus IPF group, with similar values for the breastfed reference group.

### 3.6. Adverse Events

The AST population (133 subjects in the PHF group and 134 subjects in the IPF group) was used for the safety analysis. Safety outcomes of the breastfed infants were evaluated as a reference (109 subjects). Overall, 18 serious adverse events (SAE) were reported for 12 randomized subjects (4.5%), and six SAEs were reported for four breastfed subjects (3.7%) during the study period. The majority of SAEs were in the system organ class of infections and infestations, including gastrointestinal infections, respiratory infections and ear infections. The percentage of subjects with one or more SAEs was not statistically significant different between both randomized groups, and none of the SAEs were reported to be related to study product consumption.

**Figure 3:**
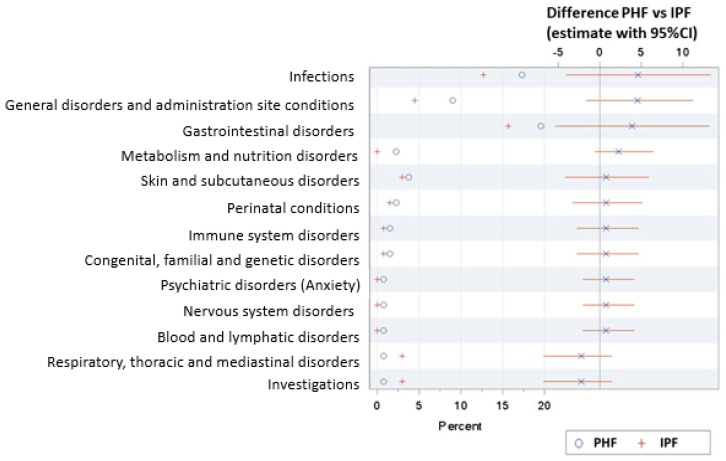
Forest plot with the estimated means as well as the difference in mean (95%) occurrence of (serious) adverse events for each system organ class per/between intervention group(s) of the AST population.

A total of 185 adverse events (AEs) were reported for 94 subjects in the randomized groups (35.2%) and 72 AEs in 37 breastfed subjects (33.9%). The most commonly observed AEs were infections (58 events in 40 subjects) and gastro-intestinal disorders (62 events in 47 subjects) in the randomized groups. In the breastfed group, there were 25 events in 19 subjects and 19 events in 11 subjects, respectively. No statistically significant differences were observed between randomized groups in the percentage of subjects with adverse events (AEs) overall or in the percentage of subjects with AEs categorized per system organ class (SOC; Figure 3). A detailed analysis per preferred term indicated that there was a higher percentage of subjects with viral upper respiratory tract infections in the PHF group compared to the IPF group (13 events in 11 (8.3%) subjects vs. two events in two (1.5%) subjects; *p* = 0.011 FE). In the breastfed group, there were three events in three subjects (2.7%). However, most events in this category were reported as “cold” or “common cold” by the investigator, and these were not considered clinically relevant, except for one subject in the PHF group with a rhinovirus infection in the upper respiratory tract who was hospitalized to allow systemic antibiotic treatment.

A higher occurrence of AEs reported as being related to the study product by the investigator was observed in the PHF group (23 events in 18 subjects) compared to the IPF group (nine events in eight subjects; *p* = 0.041 FE). Most of these were transient symptoms of functional gastrointestinal disorders, apparently randomly distributed across the wide variety of preferred terms within this SOC (e.g., diarrhea as well as constipation). All but one event, a case of atopic dermatitis in the IPF group assessed to be definitively related to study product intake, were indicated as possibly or probably related to study product intake by the investigator.

Such detailed evaluation to identify any adverse impact of the intervention products is performed as a standard procedure of clinical evaluation and serves as a precaution. Based on the assessments and adverse event outcomes described above, there was no safety concern related to (S)AEs during the study.

## 4. Discussion

This double-blind, randomized, controlled trial evaluated the safety and tolerance of a partially hydrolyzed whey-based infant formula compared to a standard intact cow’s milk protein-based formula, both containing the specific prebiotic mixture scGOS/lcFOS (9:1). An equivalent daily weight gain between enrolment and 17 weeks of age was demonstrated (primary outcome), and both study formulas supported adequate infant growth and were well-tolerated. Based on the absence of clinically relevant differences in adverse event outcomes and serum parameters between both intervention groups no safety concerns were raised.

The observed weight gain value for the PHF group in the current study is close to those reported in previous studies evaluating (partially) hydrolyzed formulas [7,17,26,27]. Previously, a randomized, clinical study of infants at risk for allergy showed that the weight development of infants consuming a whey-based partially hydrolyzed formula, which was quite similar to the PHF evaluated in the current study, was not significantly different during the first 18 months of life and only resulted in transiently higher length and head circumference values at 4 and 12 weeks compared to infants consuming a standard intact cow’s milk formula [7]. In our study, a significantly lower body weight was observed at 8 and 13 weeks of age in the PHF (and the breastfed reference group) versus the IPF group, which was not paralleled by any statistically significant differences in length or head circumference outcomes. Confirming previous findings [19], we observed infant growth outcomes for the PHF group that were close to those of breastfed infants and to the WHO growth standard median, indicative of normal infant growth. In addition, as evident from the demonstrated equivalence in daily weight gain, length gain and head circumference gain in the randomized groups, the current study confirms the suitability and nutritional adequacy of this PHF in comparison to a standard IPF, following stringent evaluation according to regulatory guidelines [20,21].

The prebiotic mixture scGOS/lcFOS (9:1), present in both intervention formulas, has been shown to stimulate colonization of the intestine with bifidobacteria which has, besides its beneficial impact on immune outcomes, a stool softening effect [28,29,30,31]. As expected, in the current study, the most prevalent reported stool consistency of formula-fed infants was “soft”. There was a low prevalence of “hard” stools and (near) absence of constipation based on reported diary information. A slightly higher prevalence of watery stools was reported for the PHF versus IPF group at 17 weeks only, which is in agreement with previous observations in preterm infants that hydrolyzed formula may shorten transit time [13]. No differences in the prevalence of diarrhea based on reported diary information or adverse events were observed between intervention groups. Moreover, the prevalence of watery stools in both formula groups remained consistently below those reported for breastfed infants throughout the study. Based on these findings, the similar volumes consumed and the similarity in other tolerance outcomes (frequency and severity of regurgitation or vomiting) between intervention groups, we conclude that the evaluated PHF is well-tolerated by infants.

In line with previous findings [7,32,33], the most common types of (serious) adverse events reported in this study are gastro-intestinal disorders and infections. Strict and detailed safety evaluation did not reveal any clinically relevant differences in the frequency, type or severity of (serious) adverse events between formula groups. A higher occurrence of AEs was reported as possibly or probably related to the study product intake as per investigator judgement for the PHF group versus IPF group. However, given the mostly transient nature and lack of specific signals (apparently random distribution across the system organ class functional GI disorders), it was not considered a safety concern. In addition, as indicated above, none of the parameters that we assessed to evaluate tolerability indicated any adverse effects of the PHF. Finally, the drop-out rate and reasons for drop-out were similar between PHF and IPF groups, with no apparent differences in the number of (S)AEs leading to product withdrawal (12.8% and 10.4%, respectively).

The voluntary blood sample obtained in a subgroup of infants at 17 weeks of age did not reveal any statistically significant differences between intervention groups. The serum urea levels are within normal ranges and the values for the subgroup of the PHF-fed infants observed in the current study (median of 18.0 mg/dL) are in line with previously reported values for 4-month-old infants consuming this PHF (median of 20.5 mg/dL) [19]. Interestingly, breastfed infants had lower serum urea levels and a lower body weight at four months of age compared to both formula groups, as reported previously [34,35], and this can be (partially) attributed to differences in protein quality and quantity [36]. Although substantial improvements in the protein quality and quantity of infant milk formulas have been made over the last few decades, the evaluated infant formulas in the current study still contained a higher protein content compared to breastmilk. Still, this study showed that the study formulas supported infant growth efficiently, given the proximity to the WHO standards, without resulting in excessive serum urea levels. However, the lower growth velocity and serum urea levels in breastfed infants suggest that there is a window of opportunity to further optimize the protein quality of the investigated infant formulas.

We consider the randomized, double-blind, controlled study design, the confirmed powered sample size and the applied high-quality standards during study conduct and evaluation to be the key strengths of this study. However, this study also has some limitations. A total of 15 study sites in six countries participated in the study. The implemented trainings and strict manuals shared with the investigators for the measurement and sampling procedures might not have fully overcome the potential increased variation in study outcomes due to the high number of study sites. Potentially, this might have contributed to the higher re-estimated required sample size during the blinded interim analysis. Conversely, one could argue that having a substantial number of study centers in a multi-country setting will increase the representativeness of the study results. Blood samples were only available for a subgroup of infants, which requires caution regarding unintended bias when interpreting these results.

In conclusion, the investigated partially hydrolyzed whey protein-based infant formula was demonstrated to support adequate infant growth, equivalent to a standard intact cow’s milk protein-based formula, and it is well-tolerated and safe for use in healthy, term infants.

## Figures and Tables

**Table 1:** Intervention product compositions ^1^.

Per 100 mL	PHF	IPF
**Fat (g)**	3.4	3.4
** Saturates**	1.6	1.5
** Monounsaturates**	1.2	1.3
** Polyunsaturates**	0.5	0.5
** Linoleic acid (mg)**	419	445
** Alpha linolenic acid (mg)**	77	83
** Arachidonic acid (mg)**	11	11
** Docosahexaenoic acid (mg)**	6.4	10.6
**Protein (g)**	1.5	1.3
** Whey protein (g)**	1.5	0.8
** Casein (g)**		0.5
**Carbohydrates (g)**	7.2	7.5
**scGOS/lcFOS (9:1) (g)**	0.8	0.8

^1^ Both formulas are iso-caloric, containing 66 kcal per 100 mL. scGOS/lcFOS (9:1), a specific prebiotic mixture consisting of short-chain galacto-oligosaccharides and long-chain fructo-oligosaccharides in a ratio of 9:1.

**Table 2:** Demographic characteristics of the per-protocol population.

	Unit	PHF (*N* = 118)	IPF (*N* = 119)	Breastfed (*N* = 98)
Sex				
Male	n (%)	53 (45%)	52 (44%)	52 (53%)
Female	n (%)	65 (55%)	67 (56%)	46 (47%)
Country				
Germany	n (%)	7 (6%)	5 (4%)	4 (4%)
Spain	n (%)	25 (21%)	25 (21%)	22 (22%)
Finland	n (%)	0 (0%)	0 (0%)	8 (8%)
France	n (%)	5 (4%)	5 (4%)	4 (4%)
Poland	n (%)	81 (69%)	83 (70%)	60 (61%)
Netherlands	n (%)	0 (0%)	1 (1%)	0 (0%)
Age at baseline (d)				
Mean age (d)	Mean (SD)	8 (4)	8 (5)	9 (4)
Birth characteristics				
Weight (g)	Mean (SD)	3360 (369)	3367 (370)	3376 (334)
Length(cm)	Mean (SD)	53 (3)	53 (3)	53 (3)
Head circumference (cm)	Mean (SD)	34 (1)	35 (1)	34 (1)
Caesarean section	n (%)	59 (50%)	59 (50%)	37 (38%)
Gestational age (wk)	Mean (SD)	39.2 (1.2)	39.2 (1.3)	39.4 (1.1)
Parental characteristics				
Maternal age (y)	Mean (SD)	29.5 (5.4)	30.7 (5.2)	31.9 (4.3)
Maternal university education (yes)	n (%)	40 (34%)	47 (40%)	71 (72%)
Maternal BMI (kg/m^2^)	Mean (SD)	24.0 (3.9)	24.8 (4.8)	23.5 (3.9)
Paternal BMI (kg/m^2^)	Mean (SD)	26.4 (3.4)	26.4 (3.0)	26.7 (3.6)

**Table 3:** Average mean (SD) daily study formula intake ^1^ during the intervention in the per-protocol population.

Formula Intake	Visit	PHF	IPF
Average daily intake, mL/d	2	618 (112)	658 (127)
	3	744 (153)	741 (152)
	4	802 (156)	784 (130)
	5	880 (183)	848 (155)
			
Average daily intake per kg body weight, mL/kg/d	2	151 (31)	157 (28)
	3	148 (30)	142 (26)
	4	133 (23)	128 (22)
	5	131 (25)	125 (24)

^1^ Reported in the 7-d diary preceding each visit. Timing of visits: V2 at 4 weeks, V3 at 8 weeks, V4 at 13 weeks and V5 at 17 weeks.

**Table 4:** Anthropometric measures of the per-protocol population during the intervention period ^1^.

Outcome Parameter	Postnatal Age	PHF # (*n* = 118)	IPF # (*n* = 119)	Breastfed Reference § (*n* = 97)
*Weight* (g)	Baseline	3393 ± 11	3396 ± 11	3423 ± 15
	4 weeks	4130 ± 20	4197 ± 20	4157 ± 24
	8 weeks	5069 ± 35 ^a^	5196 ± 34 ^b^	5060 ± 40 ^a^
	13 weeks	6075 ± 48 ^a^	6229 ± 48 ^b^	5952 ± 55 ^a^
	17 weeks	6746 ± 60 ^b^	6882 ± 59 ^b^	6475 ± 67 ^a^
*Length* (cm)	Baseline	52.2 ± 0.2	52.2 ± 0.2	52.5 ± 0.2
	4 weeks	54.4 ± 0.2	54.4 ± 0.2	54.7 ± 0.2
	8 weeks	57.3 ± 0.1	57.4 ± 0.1	57.5 ± 0.2
	13 weeks	60.6 ± 0.2	60.7 ± 0.2	60.7 ± 0.2
	17 weeks	63.0 ± 0.2	63.1 ± 0.2	63.0 ± 0.2
*Head circumference* (cm)	Baseline	34.9 ± 0.1	34.9 ± 0.1	34.9 ± 0.1
	4 weeks	36.5 ± 0.1	36.4 ± 0.1	36.4 ± 0.1
	8 weeks	38.4 ± 0.1	38.3 ± 0.1	38.2 ± 0.1
	13 weeks	40.3 ± 0.1	40.1 ± 0.1	40.1 ± 0.1
	17 weeks	41.4 ± 0.1 ^a^	41.2 ± 0.1 ^a,b^	41.1 ± 0.1 ^b^

^1^ The data are presented as means ± SE. ^a,b^ Different letters indicate significant differences between groups (*p* < 0.05). IPF = intact protein formula; PHF = partially hydrolyzed formula. ^#^ Based on parametric curve model with formula groups: (1) fixed effects: group, time (= days since birth), time^2^, sex, birth weight and country; (2) interactions: group*time, group*time^2^, sex*time and sex*time^2^; (3) random effects: intercept, time and time^2^ (for a subject) with unstructured covariance matrix. **^§^** Same model including BF group: added maternal BMI (kg/m^2^) as fixed effect for weight and for head circumference outcome analysis.

**Table 5:** Serum parameters at 17 weeks of age in the ASR population ^1^.

Serum Parameter ^2^	PHF Group (*N* = 27)	IPF Group (*N* = 38)	Breastfed Reference (*N* = 38)
Albumin, g/L	37.73 (2.89)	38.46 (2.02)	40.15 (3.00)
Calcium, mmol/L	2.66 (0.11)	2.69 (0.06)	2.72 (0.09)
Phosphate, mmol/L	2.13 (2.03; 2.27)	2.16 (2.04; 2.28)	2.03 (1.88; 2.11)
Ferritin, ug/L ^3^	97.5 (57.9; 145.0)	78.2 (61.1; 124.0)	106.0 (80.1; 170.0)
Magnesium, mmol/L	0.94 (0.05)	0.94 (0.06)	0.94 (0.05)
Urea, mmol/L	3.0 (2.5; 3.2)	2.6 (2.4; 3.2)	1.9 (1.7; 2.3)

^1^ The data are presented as mean (SD) or median (Q1; Q3). ^2^ Statistical evaluation done with *t*-test (albumin, calcium and magnesium) or Mann–Whitney test (phosphate, ferritin and urea) for comparison between PHF and IPF groups. ^3^ Samples with a C-reactive protein (CRP) value above 10 mg/L were excluded from analysis, resulting in *n* = 26, *n* = 34 and *n* = 37 for PHF, IPF and breastfed groups.

**Table 6:** Tolerance parameters in the PHF, IPF and breastfed reference group ^1^.

Parameter	Age	Severity	PHF (*N* = 133)	IPF (*N* = 134)	Breastfed (*N* = 109)
Stool frequency (n/d) ^2^	4 weeks		1.9 (1.1;3.1)	1.9 (1.3;2.6)	5.2 (3.4;6.3)
	8 weeks		1.6 (1.0;2.1)	1.3 (0.9;2.0)	2.7 (1.6;4.9)
	13 weeks		1.4 (0.9;2.0)	1.2 (0.9;1.9)	1.9 (1.0;3.6)
	17 weeks		1.4 (1.0;2.0)	1.4 (1.0;1.9)	1.4 (0.9;2.9)
Diarrhea occurrence (n, %) ^3,4^	4 weeks		8 (7%)	4 (3%)	37 (36%)
	8 weeks		5 (5%)	3 (3%)	29 (30%)
	13 weeks		4 (4%)	3 (3%)	23 (24%)
	17 weeks		7 (7%)	8 (7%)	19 (20%)
Regurgitation occurrence (n, %) ^4^	4 weeks	Occasionally	101 (82%)	101 (82%)	96 (94%)
		Commonly	48 (39%)	41 (33%)	62 (61%)
		Frequently	23 (19%)	21 (17%)	41 (40%)
	8 weeks	Occasionally	84 (76%)	94 (78%)	87 (89%)
		Commonly	32 (29%)	38 (32%)	50 (51%)
		Frequently	16 (14%)	19 (16%)	37 (38%)
	13 weeks	Occasionally	65 (61%)	79 (68%)	78 (80%)
		Commonly	30 (28%)	28 (24%)	42 (43%)
		Frequently	14 (13%)	16 (14%)	28 (29%)
	17 weeks	Occasionally	60 (56%)	68 (61%)	75 (77%)
		Commonly	22 (21%)	29 (26%)	39 (40%)
		Frequently	7 (6%)	18 (16%)	24 (25%)
Vomiting occurrence (n, %) ^4^	4 weeks	≥1 day	42 (34%)	31 (25%)	28 (28%)
		≥2–3 days	10 (8%)	9 (7%)	11 (11%)
	8 weeks	≥1 day	23 (21%)	24 (20%)	26 (27%)
		≥2–3 days	7 (6%)	5 (4%)	10 (10%)
	13 weeks	≥1 day	14 (13%)	19 (16%)	17 (18%)
		≥2–3 days	2 (2%)	5 (4%)	5 (5%)
	17 weeks	≥1 day	10 (9%)	17 (15%)	16 (17%)
		≥2–3 days	3 (3%)	5 (5%)	3 (3%)

^1^ Median (Q1; Q3) or number of infants (n) and prevalence (%) of the AST population are reported. ^2^ Stool frequency comparisons were tested using *t*-test. ^3^ Applying the WHO definition of having at least three watery stools on one day. ^4^ Occurrence of diarrhea, regurgitation and vomiting were analyzed per visit by the Miettinen and Nurminen approach to compare randomized groups. Severity of regurgitation was categorized based on occurrence and defined as occasionally (≥1 day with regurgitation), commonly (≥1 day with ≥3 regurgitations) or frequently (≥2–3 days with each ≥3 regurgitations). Statistical testing compared to breastfeeding has not been provided for any of the tolerance outcome parameters.

## Supplementary Materials

The following are available online at https://www.mdpi.com/2072-6643/12/7/2072/s1, Study Protocol TENUTO.
