# Supplementary material for: An Infant Formula with Partially Hydrolyzed Whey Protein Supports Adequate Growth and Is Safe and Well-Tolerated in Healthy, Term Infants: A Randomized, Double-Blind, Equivalence Trial
Source: Nutrients. 2020 Jul 13;12(7):2072. doi: 10.3390/nu12072072 (PMC7400250; doi:10.3390/nu12072072)
Supplement: Supplementary file 1 [file nutrients-12-02072-s001.pdf]

Study Protocol TENUTO

Ethics Statement

This study will be conducted according to the protocol and in compliance with Good Clinical Practice, the ethical principles stated in the Declaration of Helsinki, and other applicable regulatory requirements.

PROTOCOL SYNOPSIS

STUDY TITLE	A randomised, controlled, double-blind, parallel group, multi-country study to investigate the effects of an infant formula containing partially hydrolysed proteins on growth, safety, and tolerance in healthy term infants.
STUDY NAME	Tenuto
PROTOCOL NUMBER	EBB15BL89832
STUDY ARMS / PRODUCT(S)	The study consists of 2 infant formula arms i.e.:<br>• Test product: Partially hydrolysed 100 percent whey protein based infant formula containing scGOS/lcFOS.<br>• Control product: Intact cow's milk protein based infant formula (whey:casein ratio of 60:40) containing scGOS/lcFOS.<br>In addition, a breastfeeding arm is added to be used as a reference arm.
STUDY PHASE	This study is a Phase IIb study.
STUDY OBJECTIVES	To investigate the effects of a partially hydrolysed whey protein based infant formula (pHP formula) in healthy term infants until 17 weeks of age on growth, blood parameters, safety, and gastro intestinal tolerance compared to standard infant formula.
Primary	The primary objective of this study is to investigate equivalence of weight gain per day from baseline until the age of 17 weeks in infants receiving partially hydrolysed proteins (test product) compared to infants receiving intact proteins (control product).
Secondary	The secondary objectives of this study are:<br>• To investigate equivalence of weight gain per day in infants receiving formula containing partially hydrolysed proteins compared to infants receiving breast milk from baseline until the age of 17 weeks;<br>• To investigate equivalence of other growth parameters (recumbent length, head circumference, mid upper arm circumference) in infants receiving formula containing partially hydrolysed proteins compared to infants receiving formula containing intact proteins or infants receiving breast milk from baseline until the age of 17 weeks;<br>• To investigate equivalence of other growth z-scores parameters (weight-for- age, weight-for-length, length-for-age, BMI-for-age, head circumference-for-age, mid upper arm circumference) in infants receiving formula containing partially hydrolysed proteins compared to infants receiving formula containing intact proteins or infants receiving breast milk at the age of 17 weeks;<br>• To investigate the effect on blood parameters in infants receiving formula containing partially hydrolysed proteins compared to infants receiving formula containing intact proteins or infants receiving breast milk at the age of 17 weeks;<br>• To assess the effect on safety in infants receiving partially hydrolysed proteins compared to infants receiving formula containing intact proteins or infants receiving breast milk from baseline until the age of 17 weeks;<br>• To assess the effect on gastrointestinal tolerance in infants receiving formula containing partially hydrolysed proteins compared to infants receiving formula containing intact proteins or infants receiving breast milk from baseline until the age of 17 weeks.

STUDY DESIGN	This is a randomised, controlled, double blind, parallel group, multi-country, Phase IIb study.
STUDY DIAGRAM	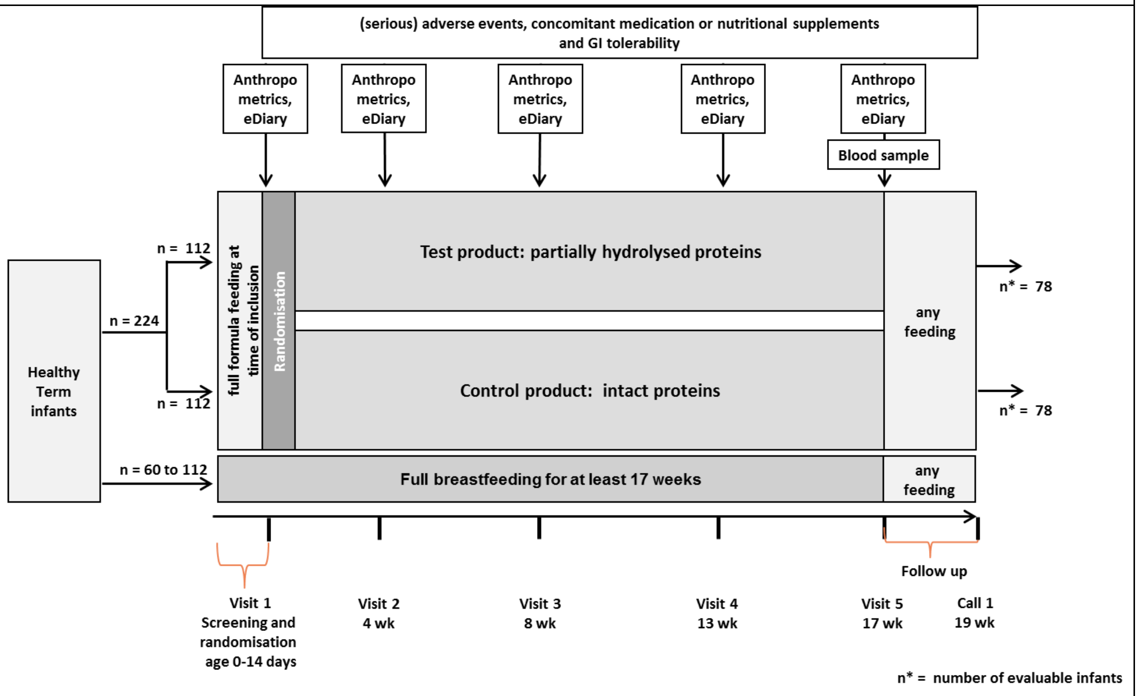
SUBJECTS	To complete this study with a total of 156 evaluable infants, approximately 224 healthy term infants will be enrolled in the two infant formula arms, anticipating a dropout rate of 30%. In addition, a maximum of 112 healthy term infants will enrol in the non-randomised breastfeeding reference arm. Recruitment for this breastfeeding reference arm stops when:<br>- Either at least 60 infants are enrolled in the breastfeeding reference arm while at the same time-point the infant formula arms are fully enrolled OR<br>- 112 infants are enrolled in the breastfeeding reference arm while at that time-point the infant formula arms are not fully enrolled yet and recruitment for these arms continues.
INCLUSION CRITERIA	1. Healthy term infants (gestational age ≥ 37 weeks + 0 days and ≤ 41 weeks + 6 days);<br>2. Infants' age at enrolment ≤ 14 days;<br>3. Birth weight within normal range for gestational age and sex (10th to 90th percentile according to the WHO Child Growth Standards – or local growth standards if available);<br>4. Head circumference at inclusion within normal range for age and sex (within 2 SD curves according to WHO Child Growth Standards – or local growth standards if available);<br>5. Infant formula arms: infants who are exclusively formula fed by time of randomisation with a maximum infants' age of 14 days (infants of mothers who choose not to breastfeed or mothers who cease breastfeeding for any reason before the infant is 14 days of age);<br>OR<br>Breastfeeding reference arm: infants who are exclusively breastfed and whose mothers are intending to exclusively breastfeed their infant at least until the infant is 17 weeks of age;<br>6. Written informed consent from parent(s) and/or legal guardian(s) aged ≥ 18 years.
EXCLUSION CRITERIA	Excluded will be:<br><u>Infants of pregnant women/mothers:</u><br>1. who are currently participating or will participate in any other (clinical) study involving investigational or marketed products during pregnancy and/or lactation;<br>2. known to have a significant medical condition (including during pregnancy) that might interfere with the study or known to affect intra-uterine growth (e.g. placenta previa, pre-eclampsia, eclampsia, gestational diabetes requiring insulin or oral medication), as per investigator's clinical judgement;

	Infants of parents:<br>3. who are incapable to comply with study protocol or Investigator's uncertainty about the willingness or ability of the parents to comply with the protocol requirements;<br>Infants:<br>4. who have to be fed with a special diet other than standard (non-hydrolysed) cow's milk based infant formula<br>5. known to have current or previous illnesses/conditions which could interfere with the study or its outcome parameters, such as gastrointestinal malformations, congenital metabolic disorders, immune deficiency or major surgery, as per investigator's clinical judgement;<br>6. with any history of, or current participation in any other study involving investigational or marketed products.
OUTCOME PARAMETERS<br>Primary	The primary outcome parameter in this study is weight gain in grams per day from baseline until 17 weeks of age.
Secondary (including safety and tolerance parameters)	Gain from baseline until 17 weeks of age of<br>• Recumbent length (mm/day)<br>• Head circumference (mm/day)<br>• Mid-upper arm circumference (mm/day)<br><br>Z-scores of anthropometric parameters at 17 weeks of age:<br>• Weight-for-age<br>• Weight-for-length<br>• Length-for-age<br>• Body Mass Index (BMI)-for-age<br>• Head circumference-for-age<br>• Mid-upper arm circumference-for-age as of 13 weeks of age<br><br>Blood parameters assessed at 17 weeks of age:<br>• Albumin<br>• Serum mineral markers: calcium, phosphorus, iron and magnesium<br>• Blood Urea Nitrogen (BUN; safety/adequacy)<br>These parameters will only be measured in blood of infants, whose parents gave additional consent to draw blood.<br><br>Safety parameters from baseline until 17 weeks of age as measured by:<br>• Number, type and severity of (Serious) Adverse events and possible relatedness to study product<br>• Use of (co-)medication<br><br>Gastrointestinal tolerance parameters from baseline until 17 weeks of age including:<br>• Occurrence and severity of gastrointestinal symptoms:<br>- regurgitation (return of the milk into the mouth without force)<br>- vomiting (return of the milk into the mouth with force)<br>• Stool frequency<br>• Stool consistency<br>• Occurrence of diarrhoea<br>• Occurrence of constipation

OTHER PARAMETERS	<u>Demographics of the infant:</u><br>• Number of infants from same pregnancy, including this infant<br>• Infant's age at enrolment<br>• Sex<br>• Race<br><br><u>Maternal and paternal medical and social characteristics:</u><br>• Age<br>• Self-reported Height<br>• Self-reported Weight<br>• BMI<br>• Relevant medical history<br>• Maternal alcohol use during pregnancy<br>• Maternal smoking habits during pregnancy<br>• Educational level<br>• First-degree relative with an allergy<br>• Allergy history parents and siblings<br><br><u>Medical information and other characteristics of the infant:</u><br>• Weight at birth<br>• Recumbent length at birth<br>• Head circumference at birth<br>• Gestational age<br>• Mode of delivery<br>• Apgar score at 1, 5, and 10 minutes after birth<br>• Medical history and prior use of medication between birth and enrolment<br>• History of defecation pattern and gastrointestinal tolerance symptoms during 3 days before enrolment (if applicable)<br>• Immunisation status<br><br><u>Feeding (history) characteristics:</u><br>• Duration of exclusive breastfeeding, derived from days between [days between birth and introduction formula/complementary feeding, whichever comes first]<br>• Age at introducing any commercial formula<br>• Age at onset of exclusively formula feeding<br>• Age at introducing study formula<br>• Age at fully formula feeding with study product, i.e. end of transition period from commercial formula to study formula<br>• Duration of mixed breast and formula feeding, derived from start and stop dates of breastfeeding and formula feeding<br>• Duration of exclusive formula feeding, derived from start of exclusively formula feeding and date end of study or date of introduction of weaning foods<br>• Use of feeding products, i.e.,<br>o Name and type of the pre-randomisation provided commercially available products<br>o Start and stop dates of study products<br>o Type and start and stop date of complementary food or milk (to check compliance)<br>• Use of nutritional supplements<br>• Amount of study product intake in week before study visit (ml/day)<br>• Type of and age at introduction of weaning foods<br><u>In case of early termination:</u>

	• Early termination date<br>• Reason for early termination
STUDY PRODUCT REGIME	Ad libitum feeding until 17 weeks of age according to local recommendation, allowing a transition period of maximally three days (approximately replacing 1/3 of the bottles per day by the study product) at start of the intervention period.<br>The breastfeeding reference group should have ad libitum exclusively breast milk until 17 weeks of age with start of weaning foods as of the age of 17 weeks at the earliest.
STUDY PERIOD	The duration of the study for each infant from enrolment to the final visit is 15-17 weeks, depending on the infant's age at study entry (max. 14 days).
STUDY DESCRIPTION	After parent(s)/legal guardian(s) have agreed to and signed informed consent, infants may enrol in the study. Infants, whose mother has the intention to breastfeed her infant until the infants' age of 17 weeks, may be enrolled in the breastfeeding reference arm. Infants, whose mother has the intention to exclusively formula feed her infant as of 14 days of age at the latest, may be randomised to one of the infant formula arms. Infants will enrol in the study as soon as they pass the eligibility check (for more details see **Error! Reference source not found.** and **Error! Reference source not found.**).<br><br>At a maximum age of 14 days all infants will have a screening/randomisation visit (Visit 1), followed by four follow up visits when the infant is 4 (Visit 2), 8 (Visit 3), 13 (Visit 4) and 17 weeks of age (Visit 5). At the infants' age of 19 weeks the Investigator performs a phone call (PC) to parents of all participating infants to follow up e.g. on safety and outstanding issues, adverse events or transition to commercial product.<br><br>During the seven days before Visit 2, 3, 4 and 5, parent(s)/caretaker(s) of all infants are requested to fill out an eDiary[1] on daily basis. The eDiary collects data on the intake of study products or breastfeeding, the infants' defecation pattern and aspects of gastro intestinal tolerance. Parent(s)/legal guardian(s) or caretaker(s) will receive automatic reminders from the eDiary system to fill out the diary.<br><br><u>Infants eligible for the infant formula arms</u><br>As soon as an infant is exclusively formula fed the screening/randomisation visit (Visit 1) takes place. At that time point, the infant will have a maximum age of 14 days.<br>After eligibility assessment, eligible infants will be randomised in a double-blinded manner to receive either the test product or the control product until the age of 17 weeks. This means that the infant receives the test or control product for a minimum duration of 15 weeks to a maximum duration of 17 weeks, depending on the age at randomisation. At Visit 1, the Investigator collects also baseline data on demographics, medical and social information, and anthropometrics. In addition, the Investigator provides the study product, gives instructions to the parents on how to use the eDiary and plans the next study visit.<br>At each of the following visits, the Investigator collects data on anthropometrics, study product intake, compliance with the study protocol, information on (Serious) Adverse Events, and information of use of medication and nutritional supplements. To support collection of data on intake of study product, the Investigator may use the data filled out by the parents in the eDiary. Likewise, data on gastro intestinal tolerance in the eDiary may support the assessment of e.g. Adverse Events related to gastro intestinal tolerance during the visits.<br>At visit 5, in addition to follow-up assessments a trained physician or nurse will draw a venous blood sample (1.4 mL) for assessment of plasma proteins and mineral status parameters, of infants whose parent(s)/legal guardian(s) provide informed consent for a blood sample on a voluntary base.<br><br><u>Infants eligible for the breastfeeding reference arm</u>

	As soon as parents or legal guardian(s) give informed consent, infants will have an eligibility assessment and enrol, if meeting the in- and exclusion criteria, in the study. The maximum age at enrolment is 14 days of age. These infants follow the same visit schedule and study assessments as the randomised infants as described above, including completion of the eDiaries and blood sampling (if separate informed consent for taking a blood sample is given).<br><br>[1] in case parents/caretakers cannot use eDiaries data will be collected via paper diaries
STATISTICAL ANALYSIS	The main statistical technique will be “repeated measurement” mixed models with randomised group as fixed factor. Sex and country are stratification factors used in the randomisation and therefore will be included in the models. Birth weight will be included as covariate in the primary model (i.e., the model on weight gain per day). The impact of factors that may influence the intervention effect may be evaluated by adding them as (time varying) covariates.<br><br>For concluding equivalence on the primary outcome parameter, the two-sided 90% confidence intervals for the differences in mean weight gain per day from baseline to 17 weeks of age will be calculated. Equivalence between the arms will be demonstrated at the 5% significance level, if the two-sided 90% confidence intervals for the differences in mean weight gain per day between the arms lies entirely between -3 g/day and +3 g/day. The margin of -/+ 3 g/day will be used if the pooled standard deviation (SD) of test and control group is equal or smaller than 6. If the pooled standard deviation is bigger than 6, an equivalence margin of 0.5xSD will be used.<br><br>Equivalence in a secondary growth parameter is demonstrated at the 5% significance level if a two-sided 90% confidence interval for the difference in mean gain per day from baseline to 17 weeks of age between test and control lies entirely between -0.5xSD and +0.5xSD. SD is the pooled standard deviation of the gain per day (in growth parameter) from baseline anthropometric visit to 17 weeks of age computed from infants receiving test and control product.<br><br>Equivalence (at week 17) in a secondary z-score growth parameter is demonstrated at the 5% significance level if a two-sided 90% confidence interval for the difference in mean z-score between test and control lies entirely between -0.5 and +0.5.<br><br>The statistical analyses for the assessment of tolerance, safety parameters, blood parameters will be descriptive.
INTERIM ANALYSIS	In order to verify whether the assumptions for the sample size calculations are correct an interim analysis will be performed when it is estimated that approximately 30 evaluable infants per infant formula arm completed the study (until Visit 5). The interim analysis will be performed on the PP data for the primary outcome parameter. In particular, the assumptions on the difference between test and control in weight gain per day from baseline to 4 months and the drop-out/major protocol violators rate will be checked. Since both study products are safe and commercially available there will be no safety interim analysis. Therefore, an independent Data Monitoring Committee will not be installed.<br><br>In order to maintain the double-blind design of the study, a study independent statistician will perform this interim analysis for checking the sample size assumptions. Based on the results of the interim analysis, an Independent Interim Committee will advise about the number of infants to be recruited. Adaptation of the number of infants to be recruited may occur accordingly. Only upward adaptation of sample size is allowed. The final sample size will be notified via an administrative amendment to the ethical committees. No further protocol changes can be based on this interim analysis.

	If the adjusted sample size becomes impractically large, the trial can be stopped for futility. Details of the interim analysis will be described in the Interim Statistical Analysis Plan, which will be finalised before the start of the interim analysis.
SPONSOR	Nutricia Research B.V., Utrecht, The Netherlands
